# Retraction: Prosthetic shape, but not stiffness or height, affects the maximum speed of sprinters with bilateral transtibial amputations

**DOI:** 10.1371/journal.pone.0280174

**Published:** 2022-12-30

**Authors:** The PLOS ONE Editors

After this article [1] was published, concerns were raised about the low sample size, whether the statistical analyses were conducted using appropriate methods, whether the results of those analyses were interpreted correctly, and whether the reported conclusions are supported by the results. In light of the concerns raised, *PLOS ONE* had the article reevaluated by an Academic Editor and a reviewer with statistical analysis expertise.

The Academic Editor advised that research on prosthetics often involves small sample sizes due to the limited population of participants meeting study criteria. They also commented that the study’s limitations were acknowledged in the article.

The reviewer advised that the article’s conclusions were not supported and that statistical non-significance was misinterpreted. The reviewer wrote, “*When a sample size is small*, *statistical non-significance does not provide sufficient evidence for a trivial effect or ‘no effect’*. *In fact*, *a non-significant observed substantial effect provides more evidence that the true effect is substantial rather than trivial*. *‘Substantial’ in this case is a change in running speed of at least 0*.*3%*, *since this change would give a top runner at least one extra medal in every 10 races*.”

The reviewer noted that Appendix Table 1 and Appendix Table 2 provide evidence that prosthesis stiffness and height both impact running speed substantially, contrary to the claims in the article’s title and conclusions.

The reviewer also advised that an assessment of the possible effects (superiority, inferiority, equivalence) of prosthesis stiffness and height can be achieved by an analysis that relies on confidence or compatibility intervals and is interpreted with reference to the smallest important changes in sprint speed (+/- 0.3%). Analyzing the data in this manner, the reviewer found that the data reported in the article [1] fall outside the confidence interval range needed to support a claim of “no effect”, both for stiffness and for height.

In light of these issues, the title and several of the article’s main conclusions are not supported, including:

- “Neither RSP stiffness expressed as a category (p = 0.836) nor as kN·m^-1^ (p = 0.916) affected maximum speed.”
- “Further, prosthetic height had no effect on maximum speed (p = 0.762).”
- “RSP shape, but not stiffness or height, influences the maximum speed of athletes with bilateral transtibial amputations.”

The authors responded to the post-publication evaluation and offered revisions to address the concerns and the reviewer’s input. They disagreed with the comment that the data do not support the article’s conclusions.

Overall, the comments and revisions received in post-publication discussions did not resolve the concerns about the data analyses or the validity of the article’s results statements and conclusions. Therefore, the *PLOS ONE* Editors retract this article.

All authors disagree with retraction.

The Amrhein 2019 article cited in the Methods is not included in the article’s Reference list. The full reference is:

Amrhein, V., Trafinnow, D., & Greenland, S. (2019). Inferential Statistics as Descriptive Statistics: There Is No Replication Crisis if We Don’t Expect Replication. American Statistician, 73, 262–270. doi:10.1080/00031305.2018.1543137
